# A Trip through Foreign Countries and Their Hospitals

**Published:** 1909-11

**Authors:** 


### Correspondence.

For Texas Medical Journal.

### A Trip Through Foreign Countries and Their Hospitals.

LETTER FROM DR. J. W. KENNEY, SAN ANTONIO, TEXAS.

A trip to a foreign country is always interesting, and to the novice fresh from his travels furnishes a theme for much talk—often to the dismay of those whom good breeding compels to listen. However, the invitation to write something of my recent trip for the "Red Back" has been accepted. Trust that my experience may be of interest to future travelers who cross the Atlantic to see what is transpiring on the other side that is of interest to the surgeon.

Having been misinformed that a long sea voyage was "good for a fellow," I selected a route covering nearly six thousand miles—beginning at Galveston, Texas, and ending at Bremen, Germany. The first stop was made at Havana, Cuba. This city was found to be one of narrow, crooked, clean streets with beautiful plazas and a splendid shell road driveway along the shore. The hotels, private homes and in fact the entire city appeared to be in perfect sanitary condition. Of the many points of interest in and about the city the Lazarus Hospital for Lepers and the General Hospital No. 1 are of special interest to medical men. The first is located in the central part of the city. It is built in the form of a hollow square and contains one hundred and fifty patients. The well kept appearance of the institution and the many different stages of the disease affecting the patients are the points attracting first attention of the visitor. Patients with the disease so far advanced that they resemble an irregular honey-combed mass of putrescent flesh and others with only a small tubercle or discolored spot on the lobe of the ear could be seen on every turn. One man, a Chinaman, had been a victim of leprosy for the greater part of his life and had been confined, chiefly in the Havana institution, for thirty-nine years. The only evidence of the disease which he presented was a small tubercle on his ear and an ulcer on one of his toes. He spoke English quite fluently and told me that his disease had always been the same—never worse nor better than at the present time. His case and that of many others in the same institution told unmistakably of the varying malignancy of leprosy and also of the slight chance one takes of contracting the disease by associating with a leper. The idea of isolating lepers and letting tubercular patients have their freedom seems preposterous—especially so when one realizes that the latter disease is far more infectious and that its toll of human life makes death from leprosy appear absolutely insignificant. As I walked about among these imprisoned patients I could not help but wonder if the few leper patients in Texas would be forced to live all the balance of their lives in mortal dread of being isolated and confined if they controlled as many votes as their more dangerously diseased consumptive brother.

The General Hospital No. 1 is located on the outskirts of the city and is arranged in the form of one-story wings connected by halls. The operating rooms and wards were up to date and presented every evidence of good management. The nurses and attendants were attentive to the patients and knew their business. The only peculiar sight about the hospital was the fact that some of the female patients were smoking big black cigars. One could almost believe the saying that all Cubans were born with a cigarette in their mouth.

As one sails away from Havana he can not help but wonder if Moro Castle was really ever seriously considered a fort and why the United States Government does not let some fisherman pull the remains of the Maine ashore with his gasoline yacht and block and tackle so that the much mooted question of whether its demise was due to an internal laceration or to an external perforation could be settled.

When about two days out on the water from Havana I began to wish for some recent work on sea sickness. Numerous remedies were suggested by considerate passengers, and I indulged in all of them from champagne to Christian Science, and still my stomach persisted in acting like a water spout on a rainy day. Rest in bed was the only relief obtainable, and in that position I remained most of the time until we landed on German soil.

After a few days spent in the northern part of Germany we reached Berlin. Here again we found an absolutely clean city—no dust or litter about the streets. The people presented a prosperous appearance and not a beggar was to be seen in the city. The chief hospitals are the Charity and the Virchow Memorial Hospitals. Each contain about two thousand beds. The Charity Hospital is of interest to the physician chiefly on account of the clinical materials to be found in it. The Virchow Hospital, said to be one of the finest in the world, is built on the cottage plan. It is of interest not only for its clinical material, but likewise on account of the handsome appearance of its grounds and buildings and the great amount of money that has evidently been expended upon and about it for the perfecting of scientific medicine. My time was spent chiefly in the operating rooms of Professor Hildebrand at the Charity Hospital and in the private gynecological clinic of Professor Leopold Landau. Each of these gentlemen operates daily and much good surgery can be witnessed at their hands. Berlin, like all large cities, has innumerable hospitals—many of them of the very best, and each numbers among its staff of physicians men with more than national reputations. Strangers are always welcome to the clinics, but must speak the German language if he expects to feel at home.

The next place visited was Vienna, Austria, which, in spite of the fact that it presents an unsanitary appearance, has long been considered the chief European medical center for American physicians. Here we find the American Medical Association of Vienna with a membership of over seven hundred. The Secretary of the Association gladly furnishes strangers all the information desired, and in a short time one feels perfectly at home. In like manner the professors and their assistants—all of whom speak English—seem to vie with each other in their efforts to make the stranger feel that he is not a stranger. Their democracy is not an artificial product, for the Austrian is by nature such, yet it is self-evident that from a business standpoint it pays. The numerous classes composed almost entirely of Americans fully substantiate this latter fact. Vienna, however, will always remain a favorite place for American medical students on account of the concentration of the hospital facilities. In the old hospital known as the Allgemeine Krankenhaus will be found sufficient clinical material to satisfy anyone on any line. The physicians of Vienna are noted for their diagnostic acumen, and the frequency with which their diagnosis are verified in the pathological laboratory gives one a splendid opportunity to judge for himself on this point. The frequency with which one meets men in this old city whose names are familiar to every physician and surgeon the world over is a very pleasant realization. Lorenz, Gersuny, Wertheim, Fuchs, Hockenegg, Werndorff, and a host of others equally as well known, are found hard at work at their post, and their reputations do not suffer upon this closer and more critical inspection. The orthopedic and plastic surgery clinics were especially interesting to me, not only on account of the vast amount of clinical material found in them, but likewise on account of the simple and common sense methods of handling such cases. The medical man is usually loath to leave vienna, and when he departs is always glad that it was his privilege to have been there regardless of the fact that during his stay he was ever conscious that his money was the chief object of all the attention given him.

After a few days spent among the beautiful mountains and lakes of Switzerland I found myself in Paris—the city heralded to the world as the center of politeness, fashion, art, literature and science. My imagination, as well as that of my wife and little daughter, had been so fed by press reports, etc., that we expected to find all these bottled up in a city with beautiful streets, handsome buildings and magnificent stores. Imagine our disappointment when in reality we found a big dirty city standing just as Napoleon left it with the exception of his own tomb, the Eiffel Tower and a poverty-stricken nobility. From an historical standpoint Paris is very interesting, but from any other view it might as well be off the map.

From Paris to London is a good move, most of the American travelers will tell you, and so I found it. London is by far the most interesting city in Europe from a medical and surgical as well as historical standpoint. The magnificent hospitals are a source of continual pleasure to the visiting physician, and the cordiality and simple manner with which the medical staff receives you makes one feel that he is more than welcome. There is a complete absence of that commercial greed among the hospital surgeons which is so evident in the other countries. The hospitals of London, like the city itself, are big—one, the London Hospital, being, figuratively speaking, as large as the city itself. It has a two hundred and fifty thousand-dollar nurses' home and employs over seven hundred nurses and has everything else in proportion. The finest hospital in the city, and, to my mind, the finest in the world, is the St. Thomas Hospital. It is new and up-to-date. The Middlesex, Guys and the hospital for sick children are, notwithstanding their age, very interesting and instructive institutions. The clinical material is overabundant, and when it looked after by such men as Lane, Bland-Sutton, Sir Victor Horsley and others of like caliber, it becomes especially interesting. More surgery or better surgery is not to be found. The original work of Lane in the treatment of fractures and auto-infection and that of Sir Victor Horsley on the brain and spinal cord are in advance of any other, while Mr. Bland-Sutton carries off the palm when it comes to ease and quickness in the performance of a major surgical operation. The absence of assumed dignity in all brainy men is especially marked in these gentlemen. Time spent in their operating rooms will be found profitable. The city of London presents many sights of historical interest, and after viewing the Old Tower, Windsor Castle, the Bank of England and a few dozen other points we left for a short trip through Ireland. The people of the northern half of this island seemed to be prosperous and contented, while those in the south were just the reverse. From a medical standpoint there was not anything of special interest, although some up-to-date hospitals are found in Belfast and Dublin. South of the latter city, however, the people are indolent and spend most of their time singing "God Save Ireland" and trying to keep out of the rain.

The start for home was made from Liverpool on the Cunard

Liner Campania. We reached New York five days later—September 17, 1909. The old saying that there is "no place like home" became a realism with me, and after spending a week in that most magnificent city in the world we started for our real home—San Antonio, Texas. Things looked better and better as we traveled toward this old city, and now when home once more there is a feeling of contentment and satisfaction that makes it more than good to be here.

There is something to be learned in every place on this globe, but there is more of it and a better quality of it to be found in the United States than in any other country. With the exception of England there is not another country that is even in competition with the United States in anything except ancient buildings and the use of press agents or their equivalents. I say this not to detract from other countries, but because of the advanced standing of the United States. There is much good in foreign countries to be gained by the physician or surgeon who may visit them, and it is a source of satisfaction to know that United States medical men are not overlooking any of it.

